# Adrenalectomy Performed with the Da Vinci Single-Port Robotic System: A Systematic Review and Pooled Analysis

**DOI:** 10.3390/cancers17081372

**Published:** 2025-04-20

**Authors:** Giuseppe Reitano, Arianna Tumminello, Carlo Prevato, Anna Cacco, Greta Gaggiato, Giorgia Baù, Lorenzo Sabato, Elisa Tonet, Anna Gambarotto, Valerio Fusca, Kevin Martina, Silvia Visentin, Giovanni Betto, Giacomo Novara, Fabrizio Dal Moro, Fabio Zattoni

**Affiliations:** 1 Department of Surgery, Oncology and Gastroenterology (DISCOG), University of Padova, 35128 Padova, Italy; 2 Department of Medicine (DIMED), University of Padova, 35128 Padova, Italy

**Keywords:** Da Vinci Single-Port, robot-assisted adrenalectomy, laparoscopic adrenalectomy, single-site surgery, adrenal masses

## Abstract

Introduction: The Da Vinci Single-Port (DV-SP) system emerged in 2018 but there is limited evidence on its use and perioperative outcomes for robot-assisted adrenalectomy (RAA). Methods: A systematic search was performed through PubMed, Scopus, Ovid, and WoS in December 2024. A PICO framework was used. Population: adult patients with adrenal masses; Intervention: DV-SP RAA; Outcomes: feasibility, reproducibility and safety of DV-SP RAA. A total of five retrospective studies involving 342 patients were included. The quantitative analysis was conducted using a random-effect model or a fixed-effect model as appropriate. A risk of bias assessment for non-randomized comparative studies and case series was performed. Results: The pooled mean operative time was 92.5 min (95% confidence interval [CI] 71.2, 113.9, *p* I^2^ = 0%, four studies), and the mean estimated blood loss (EBL) was 26.5 mL (95%CI −8.1, 61.2, I^2^ = 98.2%, three studies). Most of the procedures were completed with a single incision, though some required additional port placement, with a proportion of 9% (95%CI 0, 29, I^2^ = 71.7%, five studies). Perioperative complications were rare (0%, 95% CI 0, 4, I^2^ = 0%, five studies). Two studies comparing DV-SP and DV multi-port (MP) found no significant differences in complications. One study compared DV-SP RAA to DV Si or Xi single-access procedures. DV-SP showed improved operative techniques and better cosmetic outcomes. Limitations of this study are small sample size and potential selection bias due to smaller masses in the DV-SP RAA group. Conclusions: DV-SP RAA is a promising approach, offering reduced operative time, low EBL, and excellent cosmetic results. This study shows that DV-SP RAA seems reproducible, feasible, and safe. Limitation of the included studies are small sample size and selection bias, which limits the generalizability of the results. Randomized comparative studies between DV-SP and MP RAA are needed to further validate these findings.

## Simple Summary

Da Vinci Single-Port surgery is rapidly gaining adoption worldwide. Adrenalectomy provides an ideal context for the implementation of the Da Vinci Single-Port system. Single-port adrenalectomy appears to be both feasible and safe, potentially offering excellent cosmetic outcomes and reduced hospital stays.

## 1. Introduction

Adrenal masses encompass a variety of tumors, most of which are benign, though some can be malignant. A portion of benign adrenal masses may secrete excess hormones, but the majority (71–84%) are non-functional adenomas, often found incidentally on imaging [1]. Hormone-secreting adenomas can produce a variety of cortical and medullary hormones, including cortisol, aldosterone, and catecholamines. Malignant tumors may be metastases from other solid tumors (such as lung or kidney cancer) or primary adrenal cortical cancers. Primary adrenal cancers are rare but aggressive and present distinctive features, such as high Hounsfield units (6.3% of adrenal masses are malignant when HU >20) [2] or rapid growth (>3 mm/year) [3].

Adrenalectomy, when indicated, can be performed using either a minimally invasive technique (laparoscopy or robotic surgery) or through traditional open surgery. The latter is typically preferred for larger tumors (>6–8 cm) suspected of being adrenocortical carcinoma, as well as for those with CT evidence of local invasion [4].

Over the past 30 years, laparoscopic adrenalectomy (LA) has been the approach of choice for the surgical removal of adrenal adenomas, offering shorter hospital stay and fewer complications compared to the open approach [5]. Since its approval, the Da Vinci-Multi-Port (DV-MP) system (Intuitive, Sunnyvale, CA, USA) has been utilized for adrenalectomy, due to its ability to perform complex minimally invasive operations and to reduce the morbidity, the size and the number of incisions [6]. Robotic-assisted adrenalectomy (RAA) yields similar outcomes in terms of operative time, complications, and oncological results when compared to LA [7]. However, RAA offers enhanced ergonomics; a 3D view; tremor filtration; and superior range of motion, flexibility, and precision [8]. At the same time, RAA appears to be safer for obese patients (BMI > 30 kg/m^2^), with a lower conversion rate compared to LA [9]. Further, the robotic approach may offer a safer option for larger masses (>6 cm), as the mean operative time seems shorter with RAA compared to laparoscopic surgery [10]. However, high costs and the limited availability of robotic platforms may be significant disadvantages of RAA [9]. The development and spread of more affordable robotic platforms could present an opportunity to reduce costs and enhance the performance of RAA [11].

The evolution of robotic surgery to a single point of access may further reduce pain and potentially reduce the hospital stay [12]. The Da Vinci Single-Port (DV-SP) system uses a single arm to deliver three multi-jointed instruments and a fully wristed 3D high-definition camera through a single 2.5 cm cannula. Since gaining FDA approval in 2018, several high-volume centers have reported their experience using DV-SP for a variety of urologic procedures, including radical prostatectomy [13], simple prostatectomy [14], partial nephrectomy [15], colposacropexy [16], and pyeloplasty [17]. However, evidence regarding DV-SP use and perioperative outcomes for adrenalectomy remains limited. Significant challenges in terms of maneuverability, tissue retraction, and working space remain with the DV-SP, creating a unique learning curve compared to the DV-MP. This systematic review aims to provide a comprehensive understanding of the feasibility, reproducibility, and safety of DV-SP RAA.

## 2. Materials and Methods

### 2.1. Search Strategy

The study protocol was registered in the International Prospective Register of Ongoing Systematic Review in Health and Social Care (PROSPERO ID: CRD42023451162). In adherence with the Preferred Reporting Items for Systematic Review and Meta-Analyses (PRISMA) [18], a systematic search was conducted in December 2024 through PubMed, Scopus, Ovid, and Web of Science. The search strategy included the following terms “robot*” AND “single port” AND “adrenalectomy”. Only papers written in English were retained.

### 2.2. Inclusion Criteria, Exclusion Criteria, and Outcomes

A PICO framework was used to formulate research questions and inclusion and exclusion criteria [19]. The population of interest was composed of adult patients with adrenal masses undergoing DV-SP RAA (intervention). Laparoscopy, multiport robotic systems, and non-DV single-port devices were allowed as comparative arms (comparator). The primary outcomes of the study were feasibility, reproducibility, and safety of DV-SP RAA. Clinical trials, prospective and retrospective cohort studies, and case–control studies focusing on DV SP RAA and exploring at least one of the outcomes of interest were included. Animal models, studies on children, dry and wet lab experiences, conference abstracts, posters, editorials, and letters were excluded. Only the most recent publications were retained, and older manuscripts were excluded if data overlapped. References from the manuscript included for the full text analysis were also screened.

### 2.3. Selection Process and Data Extraction

Two authors (G.R. and A.T.) independently screened and identified relevant articles for the full text analysis using Covidence (Veritas Health Innovation, Melbourne, Australia). Data from the articles selected after the full text review were collected using Microsoft Excel (Redmond, Washington, DC, USA). The information collected was demographics, operative time, estimated blood loss (EBL), need for blood transfusions, length of stay (LoS) early and late post-operative complications according to Clavien–Dindo classification [20], postoperative pain, and positive surgical margins (PSM). Screening and data abstraction were verified by a third author (F.Z.) who was also involved in conflict resolution.

### 2.4. Risk of Bias Assessment

Risk of bias (RoB) was assessed through the ROBINS-I for non-randomized studies [21], and European Association of Urology (EAU) guidelines for systematic case series review [22] were used for the only non-comparative study. RoB assessment was carried out by two independent reviewers (A.T. and A.C.) and verified by a third author (F.Z.).

### 2.5. Statistical Analysis

A narrative synthesis of the studies was provided alongside a quantitative analysis. Pooled effects and 95% confidence intervals (95%CI) were obtained using the mean and standard deviation for continuous variables [23], while binary and categorical variables were summarized using the inverse-variance method. Event rates with 95%CI were calculated using the Freeman–Tukey double-arcsine transformation to stabilize the variance of the reported proportions. Chi-square-based Q test and I^2^ statistics were used to assess whether there was significant heterogeneity across studies. Based on heterogeneity, a meta-analysis was performed using either a random-effects model (REM, I^2^ > 50%) or a fixed-effects model (FEM, I^2^ ≤ 50%). The DerSimonian–Laird estimator was used to estimate between-study variance for binary and categorical variables. Forrest plots were used to graphically display the main results. A significance threshold of *p*-value < 0.05 was applied to determine statistical significance for the overall effect size. All analyses were conducted using STATA version 18.0 (StataCorp, College Station, TX, USA, 2023).

## 3. Results

### 3.1. Study Selection, Characteristics, and Quality

**Figure 1:**
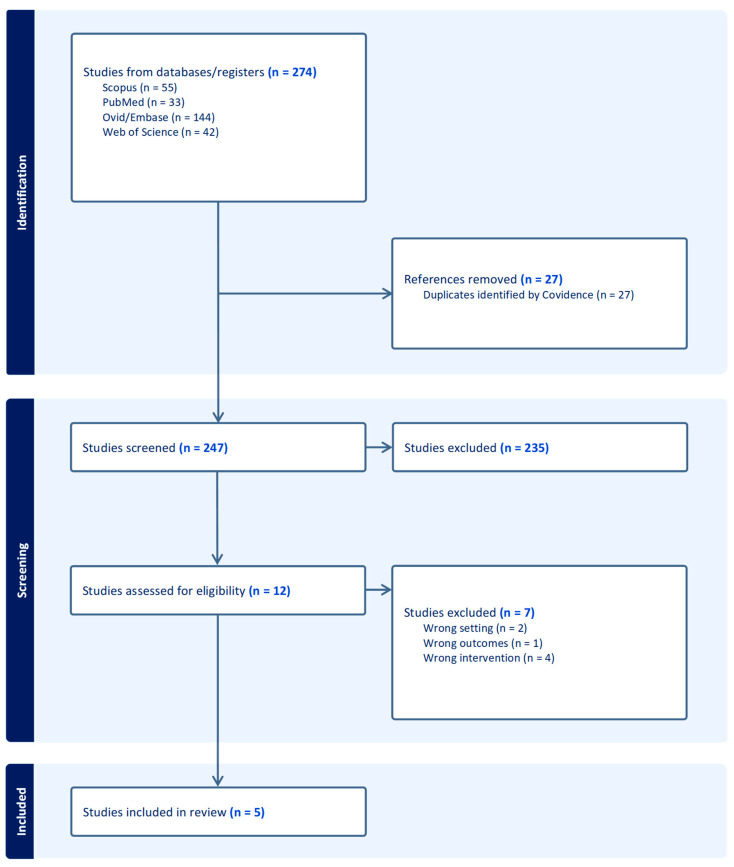
PRISMA flow-chart.

**Figure 2:**
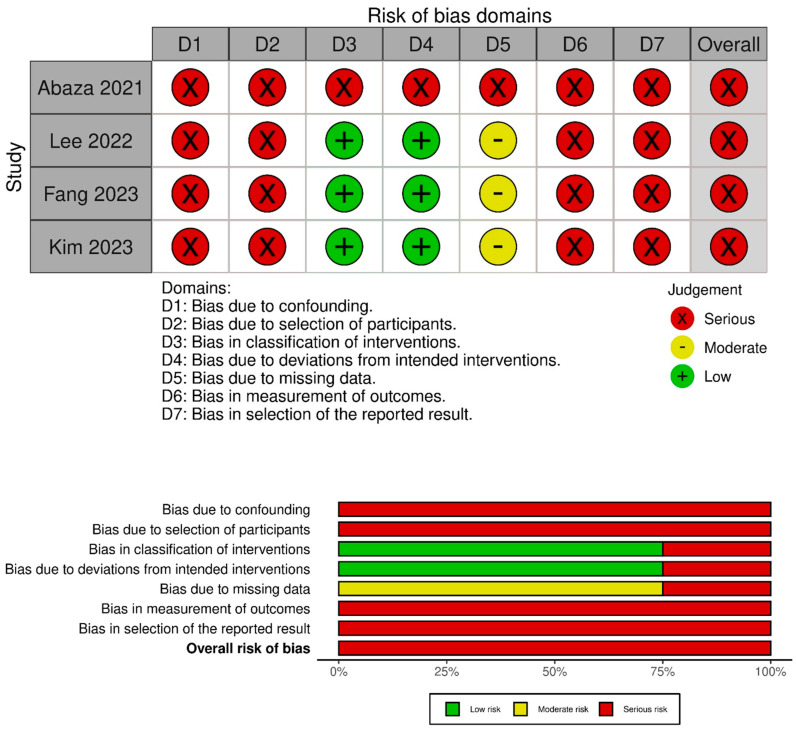
Comparative studies [12,24,25,26]—Risk of Bias Assessment (ROBINS-I).

After a systematic literature search, 274 articles were identified. Duplicates were removed either automatically using Covidence or manually. Articles that did not meet the inclusion criteria were excluded after the initial screening. Following a full-text review, five articles [12,24,25,26,27] involving 342 patients in total and 72 patients undergoing DV-SP RAA were included in the evidence synthesis (Figure 1). Only one study was excluded from the quantitative analysis of continuous variables due to aggregated data but was retained for the analysis of categorical and binary variables. According to the RoB assessment, all the comparative studies were considered to have a high-risk of bias (Figure 2). The only case-series was also at high risk of bias due to selection of participants without clear inclusion and exclusion criteria.

### 3.2. Patient Characteristics

Baseline, intra-operative, and postoperative features are summarized in Table 1. The pooled median age was 49 years (95%CI 38.7, 59.2, I^2^ = 0%), and body mass index (BMI) was 26.8 kg/m^2^ (95%CI 22.9, 30.8, I^2^ = 0%). The most common systemic syndrome associated with the presence of the adrenal mass was primary hyperaldosteronism (26/68, 38%) followed by pheochromocytoma syndrome (11/68, 16%). Incidentalomas were frequent, with 19 (28%) cases among the 4 studies included in the pooled analysis.

The pooled adrenal mass diameter was 2.2 cm (95%CI 1, 3.4, I^2^ = 0%).

### 3.3. Surgical Outcomes Associated with DV-SP RAA

Surgical outcomes can be reviewed in Table 2. Retroperitoneal DV-SP RAA was performed in 37 cases (51.4%) [24,26], while 35 (48.6%) adrenalectomies were completed using a transperitoneal approach [12,24,25,27]. Three studies [24,25,27] reported prior abdominal surgeries, accounting for a total of 23 cases.

Patients were placed either in a lateral decubitus [25], a prone jackknife position with flexion of the hip joint for retroperitoneal access [24,26], or a modified flank position with a slight flexion [27]. For the retroperitoneal approach, all authors employed a 3 cm transverse incision just beneath the tip of the 12th rib [24,26]. For the transperitoneal approach, Rudnick et al. initially used a 3 cm supraumbilical incision [27], which was later modified to a lower quadrant incision, positioned two-thirds of the way between the umbilicus and the iliac crest, while Fang et al. used a 2 cm incision on the lateral side of the umbilicus [25].

**Figure 3:**
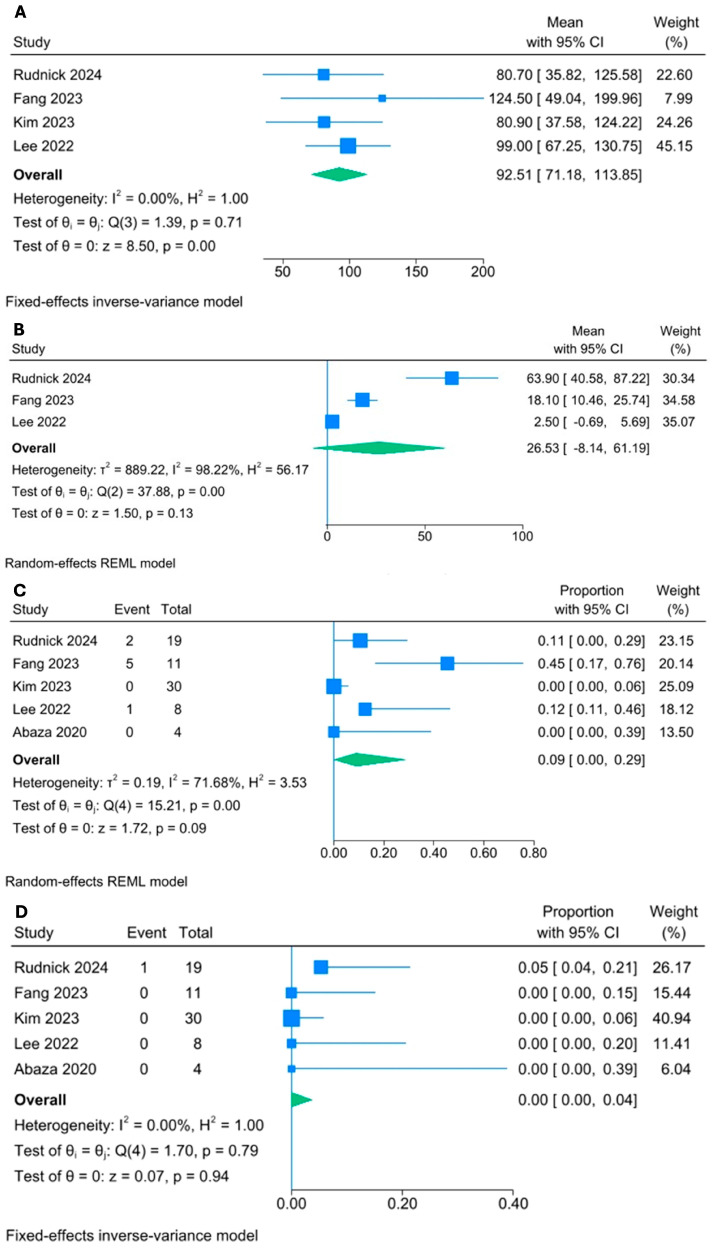
(**A**) total operative time; (**B**) estimated blood loss; (**C**) additional port placement; (**D**) complications Clavien–Dindo ≥ IIIa [12,24,25,26,27].

Pooled total operative time was 92.5 min (95%CI 71.2, 113.9, I^2^ = 0%, Figure 3A).

Most of the procedures were completed with a single incision, though some required the placement of an additional port. In three studies, an additional 5–8 mm assistant port was placed in 2 (10%) [27], 1 (12.5%) [24], and 5 (45.4%) [25] of SP cases for suction, irrigation, and retraction, and to aid in dissection. Abaza et al. [12] and Kim et al. [26] successfully performed 100% of the procedures with single access. The pooled proportion of the additional port required was 9% (95%CI 0, 29, I^2^ = 71.7%, Figure 3C).

The mean EBL was 26.5 mL (95%CI −8.1, 61.2, I^2^ = 98.2%, Figure 3B).

Conversion to open surgery or laparoscopy was not required in any case [12,24,25,26,27].

High-grade perioperative complications (Clavien–Dindo ≥ III) were rare (0%, 95% CI 0, 4, I^2^ = 0%, Figure 3D). In the study by Rudnick et al. [27], two grade II Clavien–Dindo complications (paralytic ileus and hypotension due to adrenal insufficiency managed with steroids) and one grade IIIb Clavien–Dindo complication was reported. The only high-grade complication, identified on postoperative day 6, involved a sepsis with transient ipsilateral ureteropelvic junction (UPJ) obstruction due to edema near the UPJ, which required the placement of a ureteral stent for 6 weeks. No other high-grade complications were described in the DV-SP arm [12,24,25,26].

Postoperative pain evaluated using a numeric rating scale the day of surgery was available only in two studies with means (averaged over the first six hours postoperatively) of 3.7 (0–8) [12] and 5.3 (±1.1) [26].

Same-day discharge (SDD) was achieved in 100% of the DV-SP surgeries performed by Abaza et al. [12] and Fang et al. [25]. In the other cases, LoS did not exceed 5 days [24,26,27].

PSM were reported in one patient from the series by Rudnick et al. (5.3%) [27] and one from the cases by Fang et al. (9%) [25]. Complete resection was achieved in all cases in the RAA by Kim et al. [26].

### 3.4. Comparison Between DV-SP and Two-Port or Three-Port DV-MP

Controversial results were observed in terms of operative time. DV-SP RAA seemed to offer shorter operative time in the study by Kim et al. when compared to conventional DV-MP, and this difference was also confirmed when DV-SP RAA was compared to a reduced two-port DV-MP approach (using a multiaccess glove port and a second robotic arm port) [26]. However, in the study by Fang et al., no significant differences were found in terms of operative time between the two approaches [25].

On the other hand, EBL was slightly higher in the MP group [12,25].

No significant differences in major complications were found in the comparative studies between DV-SP and DV-MP RAA [12,25]. Moreover, the only high-grade complication reported by Fang et al. was due to a pneumoperitoneum in the DV-MP arm, which required a surgical drain placement (Clavien–Dindo IIIb) [25].

In DV-SP patients, Abaza et al. [12] reported lower mean pain scores (3.7 vs. 4.5, *p* = 0.017), whereas Kim et al. did not find a significant difference in immediate postoperative pain scores (5.3 vs. 5.3 vs. 5.8, *p* = 0.958) [26].

SDD was less common among DV-MP RAA procedures in the surgeries performed by Abaza et al., with only 79% of patients discharged the same day [12]. However, among cases treated at the University of Ulsan College of Medicine, 100% of patients undergoing RAA were discharged the same day, regardless of the approach used (SP or MP) [26].

### 3.5. Comparison Between DV-SP and Single-Port Access DV-MP

Lee et al. compared DV-SP with DV-MP (Si or Xi) using a single-port access (SA) [24].

No significant differences were observed in operative and console time. Although statistical significance was not reached for docking time, a difference of 2.6 min was noted. Estimated blood loss (EBL) was similar between groups, and no perioperative complications were observed. The length of stay was 2.5 ± 0.5 days for DV-SP patients and 3.4 ± 1.1 days for SA RAA patients. Conversion to laparoscopy was required in only two (18.2%) cases among patients undergoing SA RAA, while an additional port for liver retraction was needed in the only transperitoneal DV-SP RAA case (a patient with a 3.7 cm tumor). The scar aspect was better with DV-SP RAA, as the incision size in SA-RAA patients was increased by the tension from surgical arm movements.

## 4. Discussion

The field of RAA is rapidly expanding with the introduction of several new platforms that may, in the future, offer comparable operative outcomes to the standard DV-MP [11]. In this context, DV-SP RAA, introduced in 2018, has shown feasibility, safety, and effectiveness based on early published experiences. This approach seems to yield satisfactory surgical and pathological outcomes, making it a promising advancement in the field.

Compared to SA laparoscopic and robotic surgery, DV-SP offers distinct advantages, including a flexible camera with a three-dimensional magnified view and a three-articulated, multi-jointed working arm. These features coming through a 2.5 cm cannula enhance movement efficiency and allow for more precise and fluid surgical operations. In contrast, DV-MP RAA with SA is constrained by arm collisions and often necessitates an additional port for the assistant [24]. DV-SP mitigates these issues by minimizing arm interference and reducing the assistant’s role at the operative table. A key distinction from DV-MP is that, while DV-MP relies on individual wrist rotations for each arm, DV-SP enables movement up to the wrist and elbow within the operative field.

According to a prior systematic review [28], the mean total operative time for laparoscopic RAA was 149.6 min, with a mean pooled estimated blood loss (EBL) of 85.3 mL. For DV-MP RAA, the mean total operative time was 157.2 min, and the mean pooled EBL was 66.9 mL. While a direct quantitative comparison remains challenging, our findings suggest promising performance for RAA with DV-SP.

The remarkably low mean EBL observed may be attributed to the absence of efficient intraoperative bleeding control by energy devices, necessitating meticulous dissection to prevent bleeding and precise vessel clipping. The observed reduction in total operative time may be attributed to the ease of robotic access and the reduced-port design, which generally shortens the time needed for port placement and docking, as well as smoother surgical movements with fewer collisions. However, given that early experiences with DV-SP should logically lead to longer operative time due to the learning curve associated with a new robotic platform, selection bias may have played a role in the observed efficiency. Additionally, extensive prior experience with SA laparoscopy and DV-MP may have facilitated a faster adaptation process for surgeons, allowing them to achieve proficiency with DV-SP more quickly. This was seen in the study by Kim et al. where the learning curve of DV-SP was compared to a standard three-port and a reduced two-port approach [26]. Operative time was shorter for DV-SP even in the earliest cases without a running curve during the learning process.

Also, previous experience with DV-SP may be helpful. For instance, Rudnick et al. reported the lowest mean operative time, likely due to their extensive SP robotic experience, having performed over 600 single-port robotic procedures since 2019 [27].

Regarding complications, our pooled estimate suggests a very low rate. A recent systematic review reported complication rates of 4.8% for robotic RAA and 3.9% for laparoscopic RAA [28]. In terms of LoS, DV-SP has demonstrated potential for increasing the number of same-day discharges (SDD) in selected cases, thereby reducing healthcare costs and the risk of nosocomial infections [12]. Indeed, despite offering greater safety, inpatient management is not necessary for all patients undergoing RAA [29]. Patient selection for SDD should involve a careful evaluation of the patient’s psychological and social background, especially in those requiring replacement therapy [29]. This is essential to prevent non-compliance with hormonal treatment, which can result in death or severe postoperative complications due to adrenal insufficiency [29]. Additionally, SDD should be based on a structured program of postoperative evaluations. For example, the Cosyntropin stimulation test should be performed on postoperative day 1 to assess the need for glucocorticoid replacement in patients treated for glucocorticoid-secreting tumors [30]. The program should also include office visits, telemedicine consultations, and the use of wireless sensors to monitor the patient’s vital signs outside the hospital [31].

Pooled PSM rates with DV-SP appear to be lower compared to those reported for DV-MP RAA, where the PSM rate was 11% [28]. However, the evaluation of PSM was constrained by limited available data and the predominance of highly selected small tumors, which inherently lower the risk for PSM.

From a cosmetic perspective, DV-SP may offer superior aesthetic outcomes compared to SA surgery [24,26]. As demonstrated by Lee et al., the scar appearance was more favorable with DV-SP RAA, whereas the arm movements required in MP-DV could lead to enlargement of the surgical incision [24].

Postoperative pain and analgesic use after DV-SP RAA were not consistently reported across the studies involved [12,24,26]. It is unclear whether single-port RAA can reduce postoperative pain, and this becomes even more complex when considering the use of different methods to assess postoperative pain and various painkillers, which could compromise the assessment of this outcome.

No data are currently available on disease recurrence after DV-SP RAA, so conclusions on mid-to-long-term oncological safety cannot be made.

The primary limitations of this systematic review stem from the retrospective nature of the included studies. The small sample sizes and limited number of published papers may impact the precision and reliability of the pooled estimates. Additionally, a potential publication bias due to missing information in existing literature cannot be excluded. A publication bias analysis was not performed due to the preliminary nature of the available studies. Based on the IDEAL framework [32,33], which was developed to standardize the reporting method for surgical studies, three out of four case–control studies [19,20,21] can be classified as stage 2a (development), while the experience by Abaza and the case series by Rudnick are classified as stage 1 [12,27]. The preliminary nature of the studies included justifies the low quality of the evidence. Consequently, our results should be interpreted with caution, considering the likelihood of selection bias favoring highly selected candidates for DV-SP RAA. Further research with extended follow-up durations is necessary to enhance the robustness of these findings.

To date, this systematic review and pooled analysis represent the first comprehensive assessment of surgical and early postoperative outcomes for RAA performed using DV-SP. In addition to the well-documented benefits of robot-assisted surgery, the DV-SP offers further advantages, including enhanced dexterity for surgeons due to its wide range of motion, improved intracorporeal instrument triangulation, higher rates of SDD, and better cosmetic outcomes for patients. Our findings highlight the feasibility and safety of this technique, though further investigation is needed to assess long-term outcomes. Future studies should further explore the learning curve associated with DV-SP RAA for both experienced and novice console surgeons. Larger, randomized studies are needed and may lead to a more comprehensive understanding of the platform’s clinical applications in the field of adrenal surgery.

## 5. Conclusions

This systematic review provides the most comprehensive evaluation to date of DV-SP surgery in the context of RAA, offering critical insights into its early outcomes. Despite the preliminary nature of the existing evidence and the need for further investigation, our findings affirm the feasibility, safety, and reproducibility of DV-SP RAA. The pooled data suggest that experienced robotic surgeons can seamlessly transition to DV-SP without compromising key surgical and oncological outcomes. However, the initial technical challenges and its extensive use remain important considerations. Further research is essential to delineate the full potential of DV-SP, particularly in managing complex cases such as bilateral masses and larger adenomas (>6 cm).

## Figures and Tables

**Table 1:** Baseline characteristics. BMI: body mass index; SD: standard deviation.

Author, Year	Study Design	Country	Robot	Number of Patients	Sex *n* (%)	BMI (Kg/m^2^) Mean (SD)	Prior Abdominal Surgeries *n* (%)	Adrenal Mass Diameter (cm) Mean (SD)
Rudnick 2024 [27]	Case series	USA	Da Vinci Single-Port	19	M: 10 (52.6) F: 9 (47.4)	31 (4.5)	13 (68.4)	5.2 (3.9)
Fang 2023 [25]	Case–control	USA	Da Vinci Single-Port	11	M: 3 (27.3) F: 8 (72.7)	31.8 (5.9)	8 (72.7)	2.8 (1.3)
			Da Vinci Multi-Port	25	M: 12 (48%) F: 13 (52%)	32 (8.3)	15 (60)	4.1 (1.8)
Kim 2023 [26]	Case–control	Korea	Da Vinci Single-Port	30	M: 12 (40) F: 18 (60)	24.7 (3.9)	-	2.1 (1)
			Da Vinci Multi-Port (3 ports)	117	M: 41 (35.2) F: 76 (64.8)	24.6 (3.6)	-	3.9 (2.7)
			Da Vinci Multi-Port (2 ports)	103	M: 32 (31) F: 71 (69)	25.2 (5.4)	-	3 (1.4)
Lee 2022 [24]	Case–control	Korea	Da Vinci Single-Port	8	M: 4 (50) F: 4 (50)	24.8 (3.1)	2/8 (25)	1.7 (1)
			Da Vinci Multi-Port	11	M: 4 (36.4) F: 7 (63.6)	22.2 (1.8)	3/11 (27.2)	2.3 (1.9)
Abaza 2021 [12]	Case–control	Ireland	Da Vinci Single-Port	4	-	-	-	-
			Da Vinci Multi-Port	14	-	-	-	-

**Table 2:** Perioperative outcomes. SD: standard deviation; EBL: estimated blood loss; C-D: Clavien–Dindo; PSM: positive surgical margins.

Author, Year	Robot	Total Operative Time (min) Mean (SD)	Docking Time (min) Mean (SD)	Console Time (min) Mean (SD)	Additional Assistant Port Required *n* (%)	EBL (mL) Mean (SD)	Perioperative Complications *n* (%)	Conversion to Open *n* (%)	PSM *n* (%)
Rudnick 2024 [27]	Da Vinci Single-Port	80.7 (22.9)	7.6 (2)	68 (12.6)	2/19 (10.5)	63.9 (52)	C-D < 3: 2/19 (10.5) C-D ≥ 3: 1/19 (5)	0/19 (0)	1/19 (5)
Fang 2023 [25]	Da Vinci Single-Port	124.6 (38.5)	-	-	5/11 (45.4)	18.1 (13)	C-D ≥ 3: 0/11 (0)	0/11 (0)	1/11 (9)
	Da Vinci Multi-Port	146.4 (48.1)	-	-	-	65.6 (95)	C-D ≥ 3: 1/25 (4)	0/25 (0)	0/25 (0)
Kim 2023 [26]	Da Vinci Single-Port	80.9 (22.1)	-	-	0/30 (0)	-	C-D ≥ 1: 0/30 (0)	0/30 (0)	-
	Da Vinci Multi-Port (3 ports)	134.6 (65.8)	-	-	-	-	C-D ≥ 1: 4/117 (3.4)	0/117 (0)	-
	Da Vinci Multi-Port (2 ports)	99.9 (27.6)	-	-	-	-	C-D ≥ 1: 1/103 (1)	0/103 (0)	-
Lee 2022 [24]	Da Vinci Single-Port	99 (16.2)	4.8 (2.4)	57.1 (15.2)	1/8 (12.5)	2.5 (4.6)	C-D ≥ 1: 0/8 (0)	0/8 (0)	-
	Da Vinci Multi-Port	121.9 (50.7)	7.7 (4.4)	49.1 (10.6)	-	17.3 (18.5)	C-D ≥ 1: 0/11 (0)	0/11 (0) *	-
Abaza 2020 [12]	Da Vinci Single-Port	106.4 **	-	-	0/4 (0)	-	0/4 (0)	0/4 (0)	-

* Two surgeries were completed through a laparoscopic approach. ** Data aggregated with partial cystectomy, nephrectomy, and nephroureterectomy.

## Data Availability

No new data has been created; all the data is available in the original manuscript included in the systematic review.
